# Inequalities in mortality by socioeconomic factors and Roma ethnicity in the two biggest cities in Slovakia: a multilevel analysis

**DOI:** 10.1186/s12939-015-0262-z

**Published:** 2015-11-05

**Authors:** Katarina Rosicova, Sijmen A. Reijneveld, Andrea Madarasova Geckova, Roy E. Stewart, Martin Rosic, Johan W. Groothoff, Jitse P. van Dijk

**Affiliations:** Kosice Self-governing Region, Department of Regional Development, Land-use Planning and Environment, Nam. Maratonu mieru 1, 042 66 Kosice, Slovakia; Graduate School Kosice Institute for Society and Health, Safarik University, Kosice, Slovakia; Institute of Public Health – Department of Health Psychology, Medical Faculty, Safarik University, Kosice, Slovakia; Department of Community and Occupational Health, University Medical Center Groningen, University of Groningen, Groningen, The Netherlands; Olomouc University Society and Health Institute, Palacky University Olomouc, Olomouc, Czech Republic; Faculty of Humanities and Natural Sciences, University of Presov, Presov, Slovakia

**Keywords:** Mortality, Multilevel analyses, Roma population, Socioeconomic indicators, Urban areas

## Abstract

**Background:**

The socioeconomic and ethnic composition of urban neighbourhoods may affect mortality, but evidence on Central European cities is lacking. The aim of this study was to assess the associations between socioeconomic and ethnic neighbourhood indicators and the mortality of individuals aged 20–64 years old in the two biggest cities of the Slovak Republic.

**Methods:**

We obtained data on the characteristics of neighbourhoods and districts (educational level, unemployment, income and share of Roma) and on individual mortality of residents aged 20–64 years old, for the two largest cities in the Slovak Republic (Bratislava and Kosice) in the period 2003–2005. We performed multilevel Poisson regression analyses adjusted for age and gender on the individual (mortality), neighbourhood (education level and share of Roma in population) and district levels (unemployment and income).

**Results:**

The proportions of Roma and of low-educated residents were associated with mortality at the neighbourhood level in both cities. Mutually adjusted, only the association with the proportion of Roma remained in the model (risk ratio 1.02; 95 % confidence interval 1.01–1.04). The area indicators – high education, income and unemployment – were not associated with mortality.

**Conclusion:**

The proportion of Roma is associated with early mortality in the two biggest cities in the Slovak Republic.

## Background

Inequalities in health between socioeconomic and ethnic groups are among the main challenges for public health worldwide and have been an object of study in recent decades [1, 2]. These inequalities exist at many levels – between individuals, neighbourhoods, socioeconomic groups, regions, countries and entire continents. Attempts to reduce social inequalities in health often focus on geographical disparities, since policy is most easily directed at administrative units such as local governments [3].

The number of studies treating geographical area as a separate level when studying health determinants has increased in the last two decades [3–24]. However, area inequalities in mortality within cities have been analysed much less frequently in European contexts [25–28]. This holds more for Central Europe, even though socioeconomic inequalities in health may be larger in urban areas with disadvantaged and poor populations, affecting, as a result, all city residents. Disease outbreaks, poorly maintained public places, social unrest, crime and violence are but a few of the ways that urban health inequities affect everyone. These peculiar characteristics of cities probably contribute to inequalities in health [29].

Regarding socioeconomic indicators, most multilevel studies have focused on the association between mortality and the income or education level of the population within an area [4–12, 15, 18, 24, 30–34]. Furthermore, they mostly concern Western European countries [6, 7, 9–12, 15, 17, 18], the United States [5, 24], Japan or Australia [19, 22, 23] and only recently Central Europe [35, 36]. These studies generally indicate that the income of a population within an area is a strong socioeconomic indicator of regional mortality, and that regional mortality is significantly higher in regions with larger income disparities [5, 10, 11, 24] or with lower socioeconomic status [1, 2, 23, 26, 37, 38]. Moreover, the effects of income inequality are most evident for those aged 25–64 and much stronger for males [5]. Finally, these studies have shown that the education level of the population within an area strongly correlates with the local mortality rates: people with a high education (including both sexes) have lower mortality compared with the least educated [9, 12, 31, 33, 34]. A clear picture on urban health inequalities in Central Europe is for the most part lacking.

Roma represent a large minority in the Slovak population, and they are characterised by an extremely high degree of territorial segregation, poverty and perceived discrimination [39, 40]. The health of the adult Roma population in the Slovak Republic is worse than that of the majority population. This may be due to their poor socioeconomic situation (low educational level, high unemployment rate, high proportion of poverty) and the related unsuitable living conditions and infrastructure in their places of abode, especially in the so-called settlements [39–41].

The aim of this study was to analyse the mortality of an urban population and to explore the association between area indicators – socioeconomic and ethnicity factors of the population within an area – and the mortality of individuals aged 20–64 years old on the neighbourhood level in the two biggest cities of the Slovak Republic, as an example of a Central European country.

## Methods

### Study population

The study population concerned inhabitants aged 20–64 years old in the two largest cities in the Slovak Republic – Bratislava and Kosice – in the period 2003 – 2005. The analyses were restricted to those aged 20–64 years old in order to cover the economically active population. This part of the population has the relatively lowest mortality rate, has finished the process of education and typically receives some kind of income, either in the form of a salary or as social support benefits.

In the study period the average number of inhabitants aged 20–64 years old in Bratislava and Kosice was 442,703 (47.4 % men). The total number of deaths among those aged 20–64 years old over the 3 years was 5092 (66.1 % men), i.e., a mean of 1697 per year (see also Table 1).

**Table 1:** Basic data for the Slovak population and the cities of Bratislava and Kosice for persons aged 20–64 years old – averages for the period 2003 – 2005

	Slovak Republic		Bratislava		Kosice	
	Males	Females	Males	Females	Males	Females
Standardised mortality (per 100,000 inhabitants)	616.1	242.2	485.7	225.5	563.1	252.4
Low education (elementary and without elementary)^a^	12.2 %	19.5 %	6.3 %	8.6 %	6.8 %	11.9 %
High education (tertiary)^a^	12.6 %	11.2 %	31.0 %	27.5 %	21.3 %	17.5 %
Unemployment rate	11.2 %	10.3 %	2.3 %	2.6 %	8.8 %	8.5 %
Income (whole population)	€491	€356	€705	€501	€521	€380
Roma^a^	1.4 %	1.3 %	0.1 %	0.1 %	1.6 %	1.4 %

Source: Data from the Statistical Office of the Slovak Republic

^a^Population census, 2001

### Measures

The data in this study represent three hierarchical levels: the individual, the neighbourhood and the district levels, the latter two concerning the areas where the individuals live. Individual-level data concerned the numbers of residents and the numbers of deaths, by gender and age, per neighbourhood and district of Bratislava and Kosice. Data were obtained from the Statistical Office of the Slovak Republic.

To be able to assess differences in mortality by area characteristics, we obtained data at the neighbourhood and district levels. Bratislava and Kosice are hierarchically divided into districts, which are further subdivided into neighbourhoods. Bratislava, the capital city, comprises 5 districts and 17 neighbourhoods, and Kosice, the second largest city, 4 districts and 22 neighbourhoods. The mean number of inhabitants aged 20–64 per district was 49,189 persons, ranging from 22,775 to 89,477 (average for the period 2003 – 2005). Per neighbourhood the mean number was 11,351 inhabitants, ranging from 205 to 86,722 (average for the period 2003–2005).

We obtained data on several neighbourhood characteristics: education level, unemployment rate, income and the proportion of Roma in the population of the neighbourhoods or districts. If these data were not available on the neighbourhood level (unemployment rate, income), we used data regarding the district level. The *education level* of the population within an area concerned two percentages regarding inhabitants aged 20–64 years old per area: those having no elementary education or only elementary education, and those having tertiary education. These data were based on the 2001 population census of the Statistical Office of the Slovak Republic [42]. The *unemployment rate* of the population within an area concerned the proportion of unemployed inhabitants (people not paid by an employer at all) per area aged 20–64 years old in the period 2003 – 2005 [43]. Data were obtained from the tally of the Centre of Labour, Social Affairs and Family of the Slovak Republic. The *income* of the population within an area concerned the average monthly wage of employees per area and was obtained from the Statistical Office of the Slovak Republic. Income data are available only at the district level and in the form of gross income (net income is about 75 % of gross income) and only for companies with 20 and more employees (about 60 % of all companies in the country) [44]. The *proportion of Roma* within an area concerned the percentage of the population aged 20–64 years old with Roma ethnicity per area and was obtained from the 2001 population census of the Statistical Office of the Slovak Republic [45].

#### Mortality

Data on mortality concerned the number of deaths per neighbourhood during 2003 – 2005 combined, divided into 5-year age categories (20–24, 25–29, etc.) and by gender.

### Statistical analysis

We first computed standardised mortality rates per neighbourhood, standardising by age to the total population of Slovakia. Next, we performed Poisson regression analyses in which we used mortality as the outcome and the number of inhabitants per age and gender category as the predictors. We then added the following neighbourhood characteristics to this model: education level, unemployment, income and share of Roma. We first did this for each neighbourhood characteristic separately, and we then introduced them all at once. Because of the hierarchical nature of the data – the characteristic of an area affecting all residents – we used multilevel techniques; i.e., we applied three-level Poisson regression analyses, with the levels being the individual *i*, the neighbourhood *j* and the district *k*. In these models we allowed an extra Poisson variation to estimate the level-1 (individual level) variance [46]. These analyses yielded crude and adjusted mortality risks for the various area characteristics. To assess the degree of clustering by area, we also computed random variances at the area level as well as intra class correlations (ICC).

Multilevel models provide an appropriate statistical method for describing and explaining geographic health inequalities on a range of spatial scales [21]; in other words, they allow researchers to identify ‘place effects’ on health over and above individual characteristics. The adverse health effects of area deprivation, over and above the effect due to individual SES, can only be analysed properly if the hierarchical nature of the effects is accounted for [16], i.e., characteristics of areas and communities have a potential impact on all residents [16]. Random variables at both levels were modelled to take this into account. The use of random variables at two (or more) levels of aggregation is specific for multilevel models [16].

The probability of death π of the i-th individual in the j-th neighbourhood in the k-th district was modelled as follows:

$$ \log \left({\uppi}_{\mathrm{ijk}}\right) = \log \left({\mathrm{E}}_{\mathrm{ijk}}\right) + \mathrm{constan}{\mathrm{t}}_{0\mathrm{j}\mathrm{k}}+{\upbeta}_1{\mathrm{x}}_{1\mathrm{i}\mathrm{j}\mathrm{k}}+{\upbeta}_2{\mathrm{x}}_{2\mathrm{i}\mathrm{j}\mathrm{k}} + \dots +{\upgamma}_1{\mathrm{z}}_{1\mathrm{i}\mathrm{k}}+{\upgamma}_2{\mathrm{z}}_{2\mathrm{i}\mathrm{k}} + .. + \left({\mathrm{e}}_{\mathrm{jk}} + {\mathrm{e}}_{\mathrm{k}}\right) $$

where

- log(π_ijk_) follows a Poisson-distribution with mean π (and thus also variance π)
- log(E_ijk_) is the offset of the Poisson model (i.e., the log of the expected death count for ijk)
- β_1_ represents the regression coefficient for x_1_ for the individuals in the j-th neighbourhood in the k-th district k (β_2_ for *x*_2_, etc.)
- γ_1_ represents the regression coefficient for z_1_ for the individuals in the k-th district (γ _2_ for z_2_, etc.)
- e_jk_ and e_k_ are random terms following a normal distribution, for neighbourhoods (level _j_) and districts (level _k_), respectively.

Analyses were done using SAS version 9.1., MlWin version 2.22, and SPSS version 17.0.

## Results

### Mortality

**Fig. 1:**
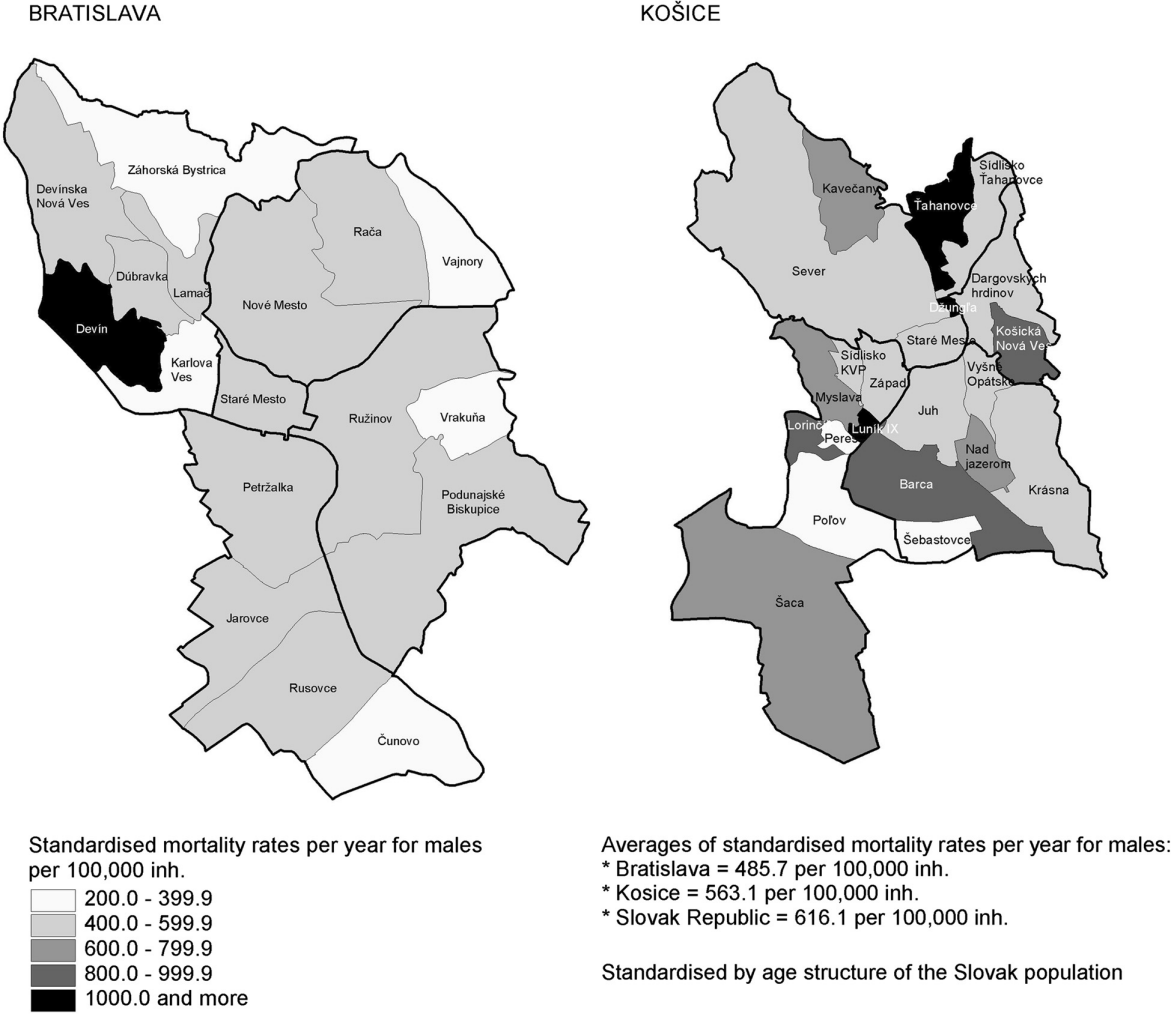
Standardised mortality rates for males aged 20–64 years by district and neighbourhood in Bratislava and Kosice. Source: Data from the Statistical Office of the Slovak Republic

**Fig. 2:**
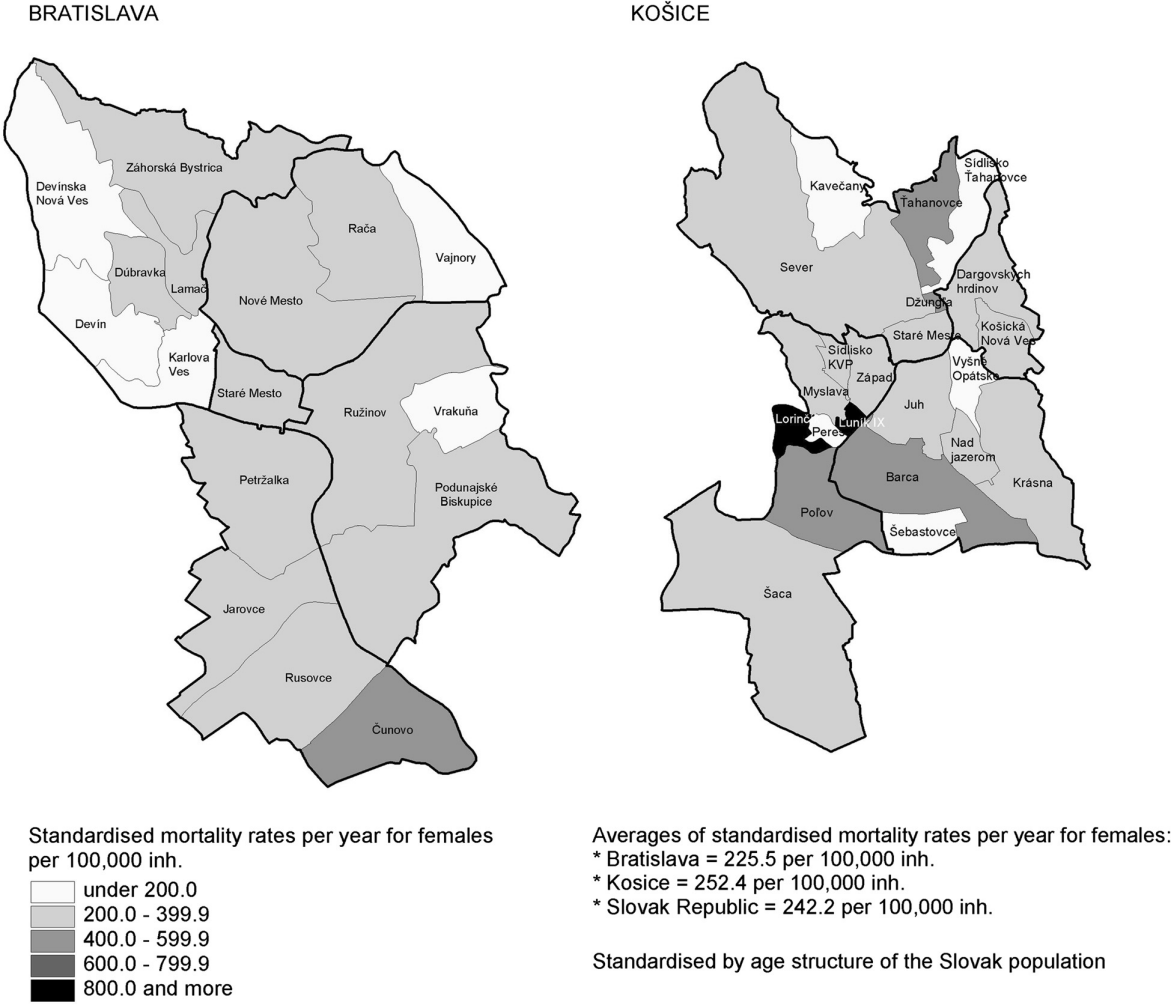
Standardised mortality rates for females aged 20–64 years by district and neighbourhood in Bratislava and Kosice. Source: Data from the Statistical Office of the Slovak Republic

In the period 2003 – 2005 the standardised mortality rates for the total population aged 20–64 years old in the Slovak Republic were 616.1 deaths per 100,000 inhabitants per year for males and 242.2 deaths per 100,000 inhabitants per year for females (Figs. 1 and 2). Regarding males, these death figures were 485.7 for Bratislava (range: 202.5 to 1665.2) and 563.1 for Kosice (range: 204.1 to 1629.8). Compared with the standardised mortality rate for males aged 20–64 years old at the national level, 10 neighbourhoods in Bratislava and Kosice (25 %) had a higher standardised mortality rate than the average rate for the Slovak Republic; 9 of these were in Kosice (Fig. 1).

Regarding females, the average standardised mortality rates were 225.5 per 100,000 for Bratislava (range: 163.9 to 587.9) and 252.4 for Kosice (range: 0.0 to 1314.3). The mortality rate for females in the examined neighbourhoods showed less marked disparities. Half of the neighbourhoods (20 out of 39) attained a higher standardised mortality rate for females aged 20–64 years old than the average national mortality rate, this being 242.2 deaths per 100,000 inhabitants per year in the study period; 14 of these were in Kosice (Fig. 2).

### Association of neighbourhood characteristics with mortality

Using multilevel Poisson regression we assessed the associations of the various area characteristics with age- and gender-adjusted mortality. In the initial crude model the degree of clustering, measured by random variance at the neighbourhood level, was statistically significant. No additional clustering occurred at the district level; therefore, this level was omitted from further analyses. Mortality risks were highest for the neighbourhood characteristics proportion of the population with low education and the proportion of Roma in the population. Introduction of the various neighbourhood characteristics led to a decrease in the random variance at the area level, i.e., a lower ICC, showing that these characteristics accounted for part of the clustering.

In the second step, we assessed the mortality risks for the two variables that led to the largest decrease in clustering for their mutual effects, mutually adjusted. This showed that only the association of mortality with the proportion of Roma within an area remained statistically significant. We did not adjust for the other characteristics because of their apparent collinearity. In this adjusted analysis, the intra-class correlation further decreased, to 0.022 (Table 2).

**Table 2:** Associations of neighbourhood characteristics with age- and gender-adjusted mortality, bivariate and with mutual adjustment: rate ratios (RR), 95 % confidence intervals (CI) and intraclass correlation coefficients (ICC)

	Unadjusted mortality risk^a^			Adjusted mortality risk^b^		
	RR	(95 %-CI)	ICC	RR	(95 %-CI)	ICC
High Education	0.992	0.986 – 0.9989	0.048			0.022
Low Education	1.010	1.004 – 1.016	0.032	1.004	0.998 – 1.010	
Income (in Euro’s)	0.999	0.999 – 1.000	0.025			
Unemployment	1.011	0.993 – 1.029	0.025			
Roma	1.028	1.016 – 1.041	0.032	1.023	1.009 – 1.037	

Source: Data from the Statistical Office of the Slovak Republic

^a^By neighbourhood characteristic, adjusted for age and gender

^b^Additionally adjusted for the other area characteristic mentioned

**Fig. 3:**
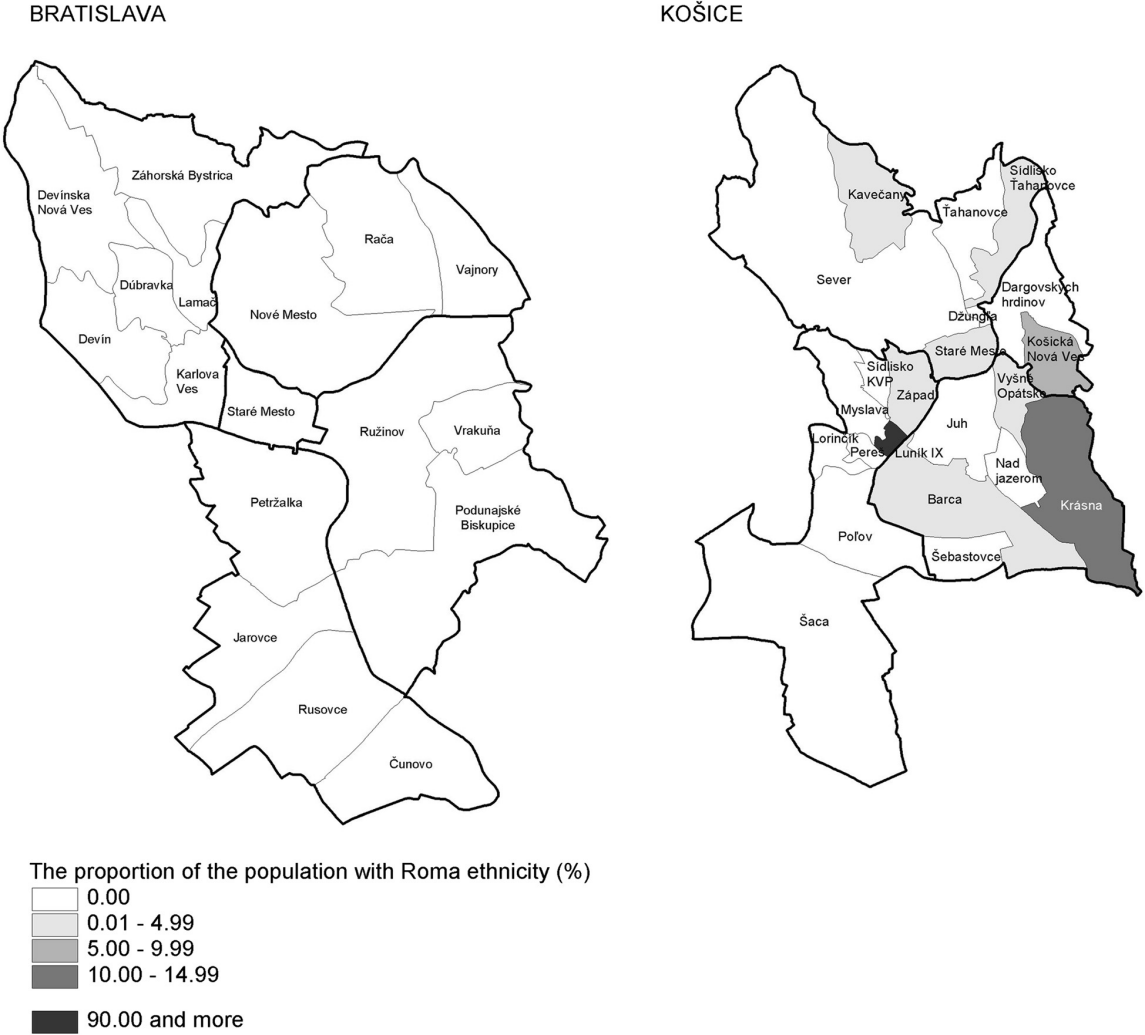
Proportion of the Roma population by districts and neighbourhoods in Bratislava and Kosice (Census 2001). Source: Data from the Statistical Office of the Slovak Republic

Figure 3 shows the spatial distribution of the Roma population (declared Roma ethnicity) by districts and neighbourhoods in Bratislava and Kosice from census data. For these two cities, the population with Roma ethnicity aged 20–64 years old is concentrated mainly in Kosice, in particular in three neighbourhoods. In the other neighbourhoods of both cities the Roma population lives largely integrated within the majority population and did not list their ethnicity as Roma in the census.

## Discussion

The aim of our study was to explore the association between socioeconomic and ethnic indicators of urban areas and the mortality of individuals aged 20–64 years old in the two biggest cities of the Slovak Republic. Our findings indicate that the proportions of Roma within an area and of those with low education per area are associated with the mortality of the urban population aged 20–64 years old. The mutually adjusted model showed that only the proportion of Roma within an area predicted the standardised mortality rate.

We found differences in mortality and in all socioeconomic indicators (education level, unemployment rate, income) and ethnicity (Roma population) between Bratislava and Kosice. These differences are based mainly on macro-spatial attractiveness (the west–east gradient), where Bratislava has a specific position due to the strong Central Europe Vienna – Budapest economic region and polarisation of the extremely developed Bratislava region (the capital city) on one hand and the rest of the Slovak Republic on the other [47]. The significant determinants of the characteristics of the Roma population are regional factors and factors regarding integration. Roma live across the whole territory of the Slovak Republic; nevertheless, there are large differences in their numbers, concentration and reproductive behaviour in different regions (48). The regions with the highest proportion of the Roma population are located in the southern part of Central Slovakia and in East Slovakia (including Kosice city) and correspond with the regions having an unfavourable socioeconomic structure [47]. The Roma population is disadvantaged by lower education levels and greater illiteracy, which represent a major barrier to successful involvement in the labour market [41].

At the neighbourhood level of Bratislava and Kosice the proportion of Roma in the population contributed to the difference in the standardised mortality rate, but in general this contribution is modest, due to a few neighbourhoods with high mortality rates and high shares of Roma. In Kosice the variation in mortality rates was generally larger and the share of Roma was higher than in Bratislava. In comparison with other studies [49, 50], our findings show a lower level of social “ghettoised” sub-urban areas in these two Slovak cities, which still have a relatively homogeneous distribution of SES across areas [35, 36]. This finding contrasts with previous findings on the regional disparities of mortality in the Slovak Republic [30, 51, 52]. Those studies showed the proportion of Roma, as an indicator of area deprivation, to be significantly associated only with infant mortality and not with adult mortality, which was significantly associated with education level or unemployment. Only a modest association between ethnicity and area mortality differences was found in the Netherlands [16], and moreover, it was found that non-Western residents seem to benefit from living in an urban environment [53].

Several studies have shown that Roma, regardless of the region they live in, have poorer health, lower life expectancy and a higher prevalence of many diseases compared with national averages or with majority populations [41, 54–57]. There are also studies that show the contribution of socioeconomic characteristics in the explanation of health differences between Roma and non-Roma [58–60]. According to the UNDP report on the living conditions of the Roma population in the Slovak Republic [41], the health of the Roma population in Slovakia is related to their poor living conditions and the poor infrastructure in their places of living, especially in settlements. In addition, Roma also have on average a very low education level, which may also contribute to their higher mortality [41, 60, 61].

### Strengths and limitations of the study

The strengths of our study are its small area design, the combination of area and individual data, the availability of age-specific population data and the perspective over time. In many countries individual-based data on mortality by age are not available, whereas area-based data are mostly available and comparable. Multilevel analysis provides a way to link traditionally distinct ecological- and individual-level studies and to overcome the limitations inherent in focusing only on one level.

The limitations of the present study are the lack of neighbourhood data on income and a potential underestimation of the number of Roma. The indicator of income (average monthly gross payment) is available at the district level in Slovakia only for companies with 20 or more employees. The share of such enterprises in the total labour market is about 60 %. As for the data on the population with Roma ethnicity, we used data from the national population census, which is based on self-identification [62]. These official censuses may underestimate the real numbers of Roma due to the tendency of Roma to denote themselves as members of more positively assessed ethnic groups [60]. A simple count of the Roma population may yield better estimates for Roma living in settlements but is not feasible for those not living in settlements. As Roma settlements occur only in Kosice and not in Bratislava, this would lead to biased regional comparisons. Therefore, we used census data because they are less biased.

### Implications

Our findings are new and thus require confirmation for other Central European countries to assess whether they are country-specific or whether they fit into a pattern which extends over several countries. Moreover, urban mortality should be analysed in relation to other health and socioeconomic factors (like social class, regional GDP and the regional Gini-index), as well as trends that occur over time.

Our results show a need to address the health needs of deprived urban areas in the Slovak Republic. These needs may be met by approaches as described by the commissioner on socioeconomic health differences [63], which are also usable in the conditions of the two largest cities in Slovakia. Improving daily living conditions is mainly a task of local government and civil society, backed by the national government, to establish local participatory governance mechanisms that enable communities and local governments to partner in building healthier and safer cities. Furthermore, local governments and civil society should manage urban development to ensure greater availability of affordable quality housing. Finally, they should plan and design urban areas to promote physical activity through investment in active transport; encourage healthy eating through retail planning to manage the availability of and access to food; and reduce violence and crime. Measuring and understanding the problem and assessing the impact of actions are mainly the tasks of research institutions and the relevant ministries. They can also help to make the social determinants of health a standard and compulsory part of training for medical and health professionals [62].

In addition, it is also important to consider interventions aimed at a revitalisation of all areas facing structural difficulties, such as e.g., proposed by EU Structural Funds. Within the framework of a policy of redistributing parts of government over the country, interventions aiming to revitalise deprived areas should focus on creating employment in such areas, e.g., by improving the developed environment, social structures (small work projects, social care, safety).

In the Slovak Republic several policies and strategies exist that focus on health, mortality, reducing regional differences and on the studied variables, including at the local level. Elaboration of these policies is an issue mentioned in the Law on regional development of the Slovak Republic, and the cities of Bratislava and Kosice have each developed such policies. Policies aimed at reducing disparities in different areas and evaluating key determinants of health can be expected to have a positive impact on the health of the population. Implementation of these policies and the application of such laws in practice is a very important step.

Regarding the Roma issue, a ‘Strategy of the Slovak Republic regarding the integration of Roma communities up to 2020’ already exists. The goal of this ‘Strategy’ is to counteract social exclusion, which occurs through the economic, cultural, symbolic and spatial exclusion of Roma communities in comparison with the non-Roma population. This policy is new; thus its impact on health will be noticeable only by thorough implementation and careful assessment of its effectiveness and by setting realistic and measurable indicators and their subsequent strict monitoring and evaluation. As Roma issues are rather complicated problems, no easy solutions are available. In the long term – a small steps approach is needed, and even then it is questionable if a not significant difference in life expectancy between Roma and non-Roma will ever be reached.

## Conclusion

The proportion of Roma was the strongest area-level predictor of urban mortality in the two biggest cities in Slovakia. A question to be answered is which concept is behind it, as other area indicators such as the proportion of inhabitants with a high education, the average monthly gross income and the unemployment rate of an area did not contribute to the prediction of mortality. Developing and implementing policies on the national, regional and local level aimed at reducing socioeconomic inequalities in mortality and addressing the health needs of the most deprived groups and the most deprived areas seems to be important for the further economic development of the country. Further research is needed to unravel the causal pathway between this particular area level predictor and socioeconomic inequalities in mortality.
